# A fluorescence lifetime-based FLIM-timer for measuring the protein turnover of transcription factor Nrf2 in live cells

**DOI:** 10.1038/s41598-025-14721-6

**Published:** 2025-08-14

**Authors:** Dina Dikovskaya, Claudia Bento-Pereira, Kanade Shiga, Andrea Corno, Maureen Higgins, Rachel Toth, Adrian T. Saurin, Albena T. Dinkova-Kostova

**Affiliations:** 1 Division of Cellular Medicine, University of Dundee School of Medicine, Dundee, DD1 9SY UK; 2 MRC Protein Phosphorylation and Ubiquitylation Unit, University of Dundee, Dundee, DD1 5EH UK; 3 Peninsula Medical School, Faculty of Health, University of Plymouth, Plymouth, PL4 8AA UK; 4 The Department of Advanced Health Science, Graduate School of Advanced Health Sciences, Saga University, Saga, Japan; 5 MRC Reagents and Services, University of Dundee, Dundee, DD1 5EH UK; 6 Department of Physiology, Pharmacology and Therapeutics, Johns Hopkins University School of Medicine, Baltimore, MD USA; 7 Department of Medicine, Johns Hopkins University School of Medicine, Baltimore, MD USA

**Keywords:** Biochemistry, Cell biology

## Abstract

Measuring protein turnover in cells has been greatly assisted by fluorescent timers (FT). However, FT quantification requires relatively high fluorescence intensity samples, prohibiting their use for proteins with low or non-uniform expression like transcription factor Nrf2, the master regulator of redox homeostasis. To visualise changes in stability/turnover of Nrf2, we constructed a genetically encoded tag combining sfGFP and mCherry and used intensity-independent Fluorescence Lifetime Imaging (FLIM) to measure Förster Resonance Energy Transfer (FRET) within the tag (named FLIM-timer). We show that the ability of mCherry to act as a FRET-acceptor develops as the protein matures, allowing the use of FLIM-FRET as a readout of the FLIM-timer. FLIM-timer-tagged Nrf2 allowed to observe differences in its turnover between cellular compartments with equal precision in regions of high and low brightness. The reduction in fluorescence lifetime of FLIM-timer-Nrf2 confirmed its stabilisation by sulforaphane. Depletion of a degron for either Keap1-Cul3 or SCF^β-TrCP^-mediated degradation decreased the fluorescence lifetime of Nrf2-FLIM-timer. FLIM-timer labelled cyclin B was also successfully used to track its destabilisation during mitotic exit. Thus, FLIM-timer methodology increases the FT applicability for visualisation and quantification of protein turnover, expanding it to cells with low and variable levels of any protein of interest.

**Supplementary Information:**

The online version contains supplementary material available at 10.1038/s41598-025-14721-6.

## Introduction

**Fig. 1:**
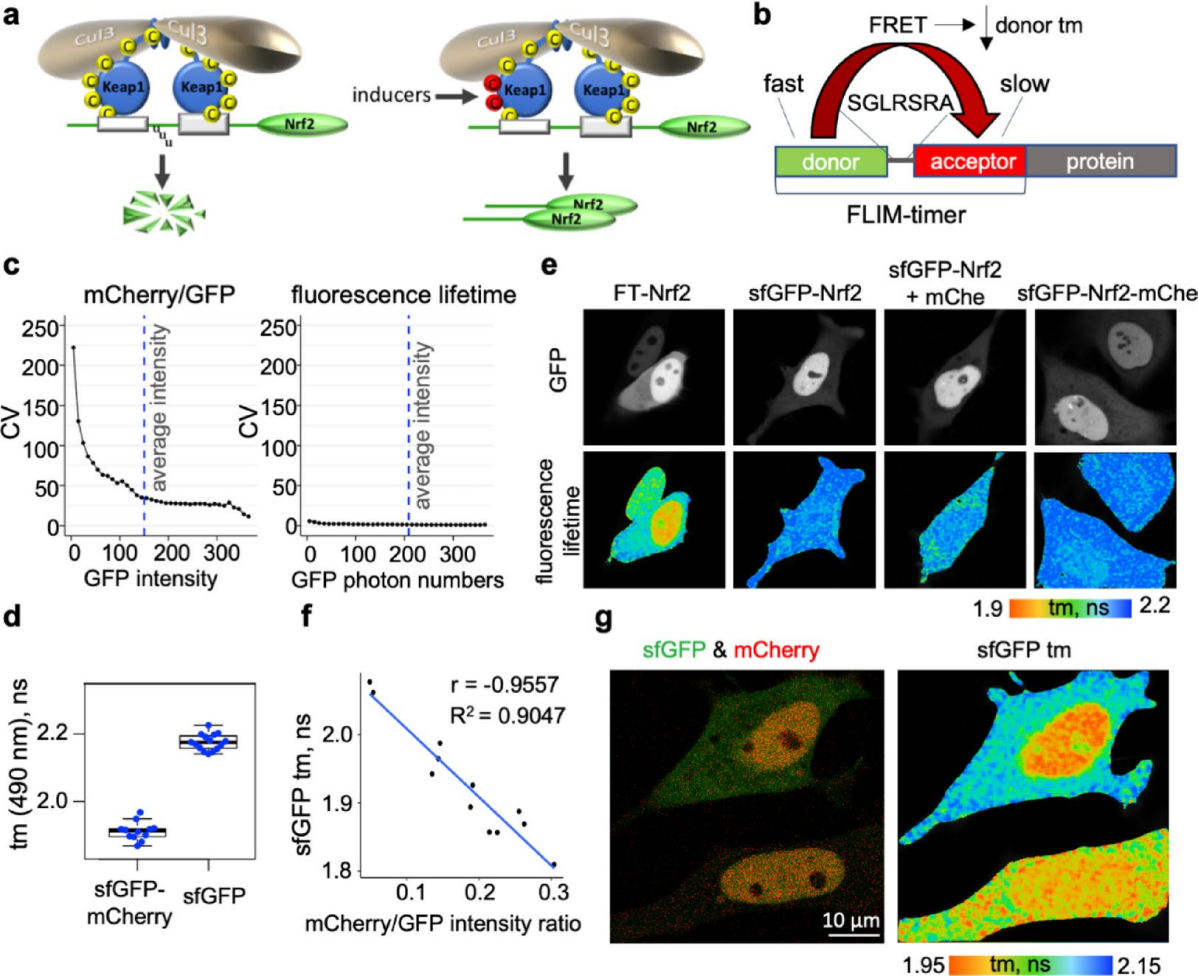
FLIM-based fluorescence timer for measuring Nrf2 turnover. (**a**) Nrf2 regulation by Keap1. At basal conditions (left), Nrf2 is targeted for proteosomal degradation by Cullin3–RING ubiquitin E3 ligase in a complex with its adaptor Keap1 that recruits Nrf2. Modifications of sensor cysteines in Keap1 by inducers ROS or electrophiles (right) inhibit Keap1-Cullin3-mediated degradation of Nrf2, stabilises Nrf2 and initiates oxidative stress response. (**b**) Design of a FLIM-timer protein tag, composed of the fast-maturing FRET donor and the slower maturing FRET acceptor, linked by a linker peptide. FRET between tag components shortens the fluorescence lifetime (tm) of the FRET donor and serves as readout for protein turnover/stability. (**c**) The pixels within cellular areas shown and quantified in Suppl Fig 1a-c were grouped by their GFP channel intensities into 37 equal intensity bins, and the Coefficient of Variation (CV) for mCherry/GFP ratio (left) or fluorescence lifetime (right) calculated for each bin was plotted against mean bin intensity. Blue dashed lines show average values of cellular area. (**d**) Fluorescence lifetimes sfGFP-mCherry FLIM-timer and the sfGFP control constructs in Dox-induced HeLa-FRT/TO cells is measured using InTune laser. Fluorescence lifetime (tm) determined in entire cellular areas using global binning as described are shown as dots, boxes outline the data range within lower and upper quartiles, thick line marks median and whiskers extend to the highest and lowest values excluding outliers. (**e**) Photon numbers (top panels) and fluorescence lifetime heat maps (bottom panels) of HeLa cells transiently transfected with sfGFP-mCherry-Nrf2 (FT-Nrf2), sfGFP-Nrf2 with or without co-transfection with free mCherry, or sfGFP-Nrf2-mCherry, as is indicated. The colours correspond to the tm values as shown underneath. Representative images from 7, 3, 7 or 10 FLIM datasets respectively, are shown. Quantification in Suppl. Fig 2b. (**f**) Strong negative correlation between ratiometric and FLIM-based measurements shown by Pearson Correlation Coefficient (r) and a slope of the linear fit of the data with the high goodness of fit (R^2^). Dots are globally measured values of fluorescence lifetime (tm) and GFP fluorescence intensities ratios measured in the same nuclear or cytoplasmic regions (N = 12) of HeLa cells transiently transfected with sfGFP-mCherry-Nrf2. Fluorescence lifetime acquired with 2-photon laser, followed by confocal imaging. (**g**) Confocal image of HeLa cells transfected with FT-Nrf2 displayed as an overlay of sfGFP (green) and mCherry (red) channels (left panel) or a fluorescence lifetime map obtained with 2-photon laser viewing FLIM (right). The false colours correspond to the tm values as shown underneath. See also Suppl Figs. 1 and 2.

Transcription factor Nuclear Factor Erythroid 2-related Factor 2 (Nrf2) drives the cytoprotective transcriptional program that is activated in response to oxidants and electrophiles. In homeostatic conditions, Nrf2 is an unstable protein with a half-life of about 20 min^1,2^. It is continuously targeted for degradation by the Keap1-Cullin3-RBX1 E3 ubiquitin ligase complex, with Keap1 acting both as E3 adaptor for Nrf2 and a redox sensor (Fig. 1a). Activation of Nrf2-mediated oxidative stress response is achieved by fast stabilisation of Nrf2 by oxidants or electrophiles, collectively known as Nrf2 inducers. Nrf2 inducers modify specific reactive cysteines in Keap1, leading to an inhibitory change in the Nrf2-Keap1-Cullin3 complex confirmation^3,4^. This results in Nrf2 accumulation, followed by nuclear translocation of Nrf2 and transactivation of Nrf2 target genes.

Induction of Nrf2 allows for adaptation and survival under stress conditions and has shown protective effects in many preclinical models of human disease and in clinical trials. A growing number of pharmacological Nrf2 inducers are in various stages of drug development to counteract oxidative stress, toxicity and inflammation in chronic diseases^5,6^. On the other hand, in several cancer types, excessive Nrf2 activation due to mutations disrupting Nrf2 degradation pathways promotes chemoresistance and is pro-tumorigenic^7^. While early attempts to develop specific Nrf2 inhibitors were not successful, the PROTAC and molecular glue technology now offers a new avenue to target Nrf2 in cancers^8^. The development of both inducers and degraders of Nrf2 require a reliable assessing technique.

Several methods have been developed to assist identification and validation of potential Nrf2 inducers by reporting on Nrf2 transcriptional activity or its interaction with Keap1 (reviewed in^9^). Here we developed a novel approach to measure activation of Nrf2 by monitoring Nrf2 stabilisation with the use of a modified fluorescent tag. In contrast to previously used target gene expression reporter assays, this approach detects an early step in Nrf2 induction. Furthermore, unlike assays that detect modifications of Keap1 or the Keap1-Nrf2 complex, it is not biased towards Keap1-mediated regulation and can report on the modulation of any of the Nrf2 degradation pathways^10–12^, which makes it also perfectly suitable for monitoring activities of PROTAC-based Nrf2 degraders. This work validates our new approach to monitor protein stability that can be further applied to any number of molecular targets.

## Results

### Generation of FLIM-timer as a readout for protein turnover

To monitor Nrf2 stability in real time, we considered to employ a Fluorescent Timer (FT), a fluorescent molecule that changes its spectra over time to allow assessment of protein turnover and trafficking within live cells or an organism at cellular and subcellular resolution^13–16^. Traditionally, the stability of FT-tagged protein can be inferred from the amount of fluorescence measured at the wavelength that corresponds to the converted (late) FT, relative to the fluorescence intensity in the initial FT spectra. Thus, the ratio between converted and initial fluorescence intensities serves as FT readout, with low values indicating high turnover, and vice versa. However, the ratiometric nature of the measurements makes it difficult to apply FT method to the targets with low or strongly variable expression levels such as Nrf2, because FT quantification loses its precision at low initial FT intensity used as a denominator. To overcome this limitation, we decided to develop an alternative protein turnover measurement technique based on fluorescence lifetime. Fluorescence lifetime is the average time that a fluorophore spends in the excited state before returning to the ground state by the emission of a photon. Furthermore, the fluorescence lifetime is independent of fluorophore concentration and laser intensity^17^.

For this purpose, we constructed a genetically encoded protein tag composed of two fluorophores capable of Förster Resonance Energy Transfer (FRET) between them, connected by a short linker that positions the fluorophores in a way that promotes FRET^18,19^ (Fig. 1b), and employed Fluorescence Lifetime Imaging (FLIM) to measure FRET between the tag components. FRET reduces fluorescence lifetime of the fluorophore acting as a FRET donor, and this reduction is independent of the amount of the FRET donor and proportional to the FRET efficiency^20,21^. Consequently, fluorescence lifetime measurements using FLIM provide a highly sensitive and quantitative readout for FRET.

To confirm that this method is more consistent across variable intensities than ratiometric measurements, we compared fluorescence lifetime measured in cells transfected with FRET-capable EGFP-mCherry construct, to that of the ratio between mCherry and EGFP intensity in the same cells (Suppl Fig. 1a), across the entire range of pixel intensities. Consistent with the notion that fluorescence lifetime is largely independent of the level of FRET donor, the average EGFP fluorescence lifetime was similar among pixels with low and high intensities (Suppl Fig. 1b, dark blue line in the inset). In contrast, the average mCherry/GFP intensity ratio in the same cell was higher for pixels with low GFP intensity, likely because of the effect of high dispersion near zero boundary (Suppl Fig. 1c, dark blue line in the inset).

The average coefficient of variability (CV) for ratiometric measurements was 46.77% (32.56% for pixels with intensities above background), and it increased sharply in pixels with low intensity (Fig. 1c, left), as expected. In contrast, the variability of fluorescence lifetime among pixels was overall much lower, with mean CV equal 1.99% (1.45% for pixels with intensities above background). Importantly, there was very little, if any, difference in fluorescence lifetime variability among dim and bright pixels of the image (Fig. 1c, right). This confirmed that FLIM-FRET measurements are more reliable than ratiometric intensity measurements in systems with non-uniform or low expression of fluorescent proteins.

To achieve the FT-like time-dependent changes in FLIM-FRET, we took advantage of the previously developed concept of tandem fluorescent timer (tFT)^16^ that combines fluorophores with different maturation times, i.e. the times during which the newly synthesized fluorophores are spontaneously rearranged to become fluorescent^22,23^. In this system, the difference between the onset of fluorescence for the two tag components creates a time window in which the intensity of the fast-maturing fluorophore is constant, while the intensity of the slow-maturing fluorophore increases over time, changing the ratio between their fluorescence intensity with time, similar to that observed in single-molecule FT. We hypothesised that the ability of a fluorophore to accept FRET is also dependent on its maturation state. If our hypothesis is correct, the combination of a FRET donor with fast maturation and a FRET acceptor with slow maturation (Fig. 1b) should lead to a minimal intra-tag FRET in the newly synthesized protein which then will increase over time as a result of FRET acceptor maturation. Consequently, the fluorescence lifetime of FRET donor within such tag (referred further as FLIM-timer) could serve as readout for protein stability, with its highest values corresponding to high turnover/unstable protein, and vice versa. In other words, if the protein degradation is faster than the maturation speed of the FRET acceptor, than a certain proportion of molecules will be degraded before FRET could occur, reducing the overall FRET efficiency and keeping the fluorescence lifetime of a donor fluorophore high.

To test this idea, we selected sfGFP^24^ that has fast intrinsic maturation as FRET donor, and mCherry, which has a slower maturation kinetics, as a FRET acceptor^23^, a fluorophore combination previously used for tFT in yeast^16,25^. The maturation times for these fluorophores have been reported in yeast (5–7 min for sfGFP, and between 22 and approximately 40 min for mCherry^16,26^) and in e.coli (14 min for sfGFP and between 23 and 37 for different versions of mCherry^23^). We confirmed the efficient FRET between sfGFP and mCherry, as seen by a strongly reduced fluorescence lifetimes of sfGFP in the context of the tag, compared to that without mCherry (Fig. 1d), with the average FRET efficiency 12.13%.

Since the robust estimation of fluorescence lifetime using Time-Correlated Single Photon Counting (TCSPC) imposes the lower limit on the number of photons per pixel^27^, we selected to use the 1-component model to analyse all data that requires the least numbers of photons and thus limits duration and/or intensity of illumination. We have previously shown the validity of such approximation for the relative FLIM-FRET measurements in live cells^19^. The use of 1-component fit generated robust data with uniform residuals, though it has slightly reduced the goodness of fit, compared to the 2-component fit (Suppl Fig. 1d). The difference between the maximal and minimal fluorescence lifetime was approximately 350 ps, providing sufficient resolution for monitoring changes in protein stability using FLIM. Note that the intrinsic turnover rate of such composite tag is much slower than the maturation rate of mCherry, therefore on its own it could be considered a stable protein.

To determine whether the fluorescence lifetime of such tag can report on changes in protein stability of Nrf2, we generated a fusion of sfGFP-based FLIM-timer with Nrf2. N-terminally FLIM-timer-tagged Nrf2 overexpressed in HeLa cells localised predominantly to the nucleus (Fig. 1e), as previously observed for a transiently transfected fluorescently-labelled Nrf2^19^. Our previous work confirmed that N-terminally tagged Nrf2 remains transcriptionally active and retains Keap1-dependent regulation^3,19^. Comparison of one- versus and two-component models for fluorescence lifetime in such construct showed that the 2-component fitting made only a very small improvement in the goodness of fit (from R2 = 0.9978 to R2 = 0.9996) (Suppl Fig. 2a), therefore in order to reduce the required number of photons and limit illumination of live cells, 1-component analysis was used in most experiments. The fluorescence lifetime of nuclear FT-Nrf2 was much shorter than that in the cytoplasm (left bottom panel in Fig. 1e), indicative of more stable Nrf2 protein in the nucleus than in cytoplasm. The reduced nuclear fluorescence lifetime was unique to FT-Nrf2 and not observed for sfGFP-Nrf2 expressed with or without free mCherry. Interestingly, Nrf2 tagged with sfGFP at its N-terminus and with mCherry at its C-terminus, the tagging strategy that can act as ratiometric fluorescence timer but not as FLIM-timer, has shown a slight but significant reduction of fluorescence lifetime in the nucleus (Suppl. Figure 2b), which could potentially result from Nrf2 folding or the presence of higher-order structures that promote FRET between N- and C-termini of Nrf2 either in cis (within the same molecule) or in trans (between different Nrf2 molecules).

Remarkably, mCherry/sfGFP intensity ratio (a previously used measure for stability of tFT-tagged proteins) in sufficiently bright areas of cells transiently transfected with this construct strongly negatively correlated with the fluorescence lifetime (denoted further as tm) of the FRET donor sfGFP (Fig. 1f). This is consistent with maturation-dependent changes in FRET efficiency of the FLIM-timer and confirms that fluorescence lifetime of FLIM-timer-tagged proteins can be used as readout of their turnover. Similar results were obtained for fluorescence lifetime calculated with 2-component fitting (Suppl Fig. 2c). The strong correlation with high goodness of fit was also apparent between mCherry/sfGFP intensity ratio and FRET efficiency of FLIM-timer (Suppl Fig. 2d). FLIM also improved visualisation of stability/turnover of FLIM-timer-tagged Nrf2 overexpressed in HeLa cells (Fig. 1g), as it produced a high-contrast map in regions of both high and low levels of tagged protein.

### Monitoring drug-induced Nrf2 stabilization

**Fig. 2:**
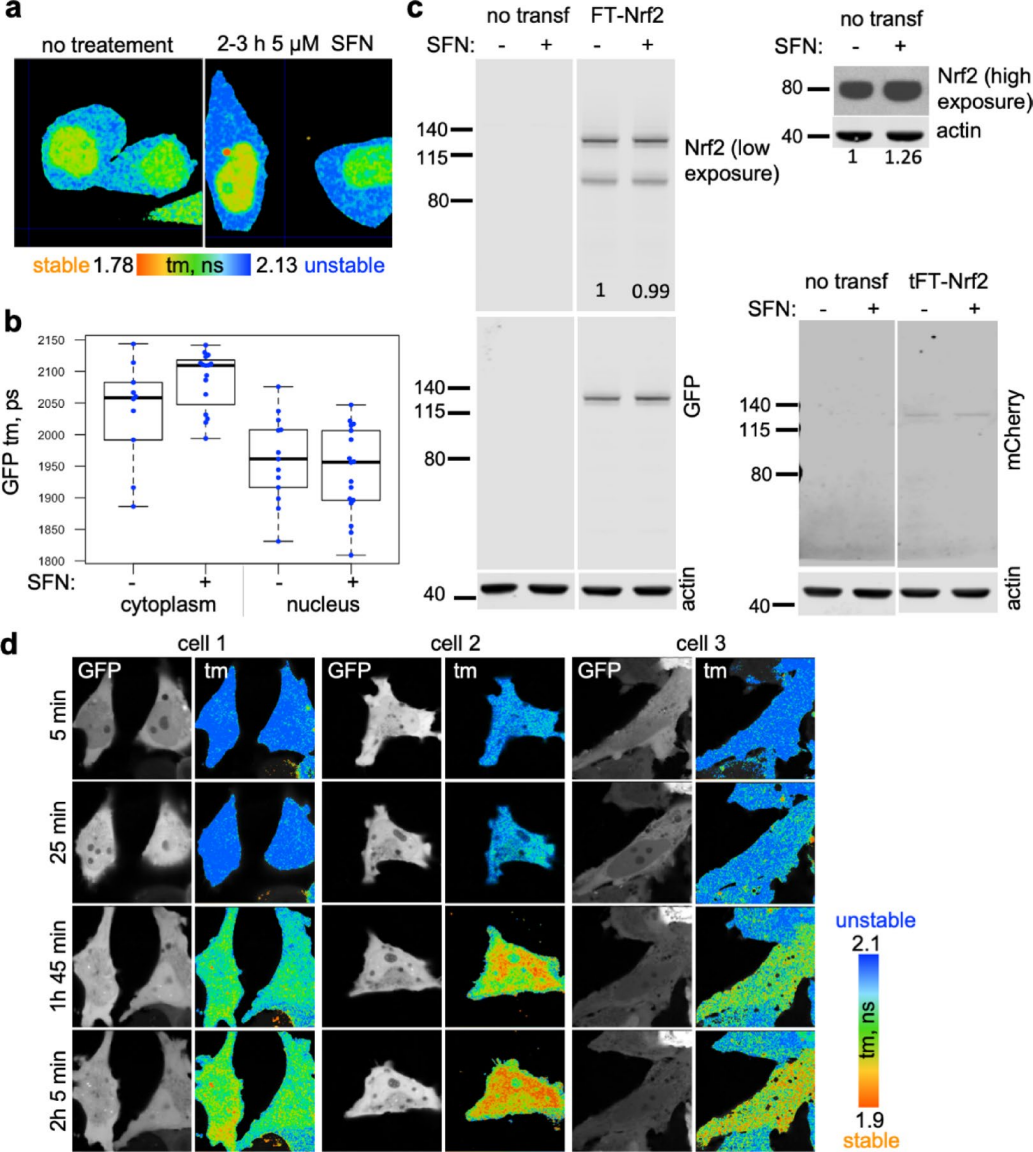
Visualisation of Sulforaphane-induced stabilization of Nrf2 using FLIM-timer.(**a**) HeLa cells transfected with FT-Nrf2 (sfGFP-mCherry-Nrf2), before (left) and after (right) 2–3 h treatment with 5 µM sulforaphane (SFN). The colour coding for the fluorescence lifetime is shown underneath. (**b**) Quantification of a. Each dot represents mean fluorescence lifetime within either nucleus or (tm) cytoplasm, and the box plots summarise data distribution as in Fig. 1d. (**c**) Untransfected or FT-Nrf2-transfected HeLa cells were treated for 1 h with 5 µM SFN or left untreated. 10 µg of lysates were immunoblotted for Nrf2 (top left), GFP (bottom left) or mCherry (bottom right). 20 µg of lysates from un-transfected cells were re-blotted for Nrf2 followed by an extended ECL detection (top right). Actin is a loading control. Relative Nrf2 levels normalised to actin are shown under the blots. Note that SFN stabilises endogenous but not endogenously overexpressed Nrf2. Full blots are in Supplementary Information. (**d**) Photon numbers (left panels, GFP) and fluorescence lifetime heat maps (right panels, tm) in three HeLa cells co-expressing FT-Nrf2 and Keap1, over the 125 min course of treatment with 5 µM SFN. The colour coding for the fluorescence lifetime is shown on the right. Quantifications are in Suppl Fig. 2e. See also Suppl Fig. 3.

We next wanted to see whether FLIM-timer-tagged Nrf2 can detect Nrf2 activation by an inducer, by reporting on stabilisation of Nrf2 protein. Surprisingly, treatment of cells with an established Nrf2 inducer sulforaphane (SFN) that targets Keap1^5^ did not stabilise the overexpressed FT-Nrf2 (Fig. 2a–c), though the level of endogenous Nrf2 was increased by the same treatment (Fig. 2c, top right panel). One possible explanation for this result is that visible amounts of overexpressed FT-Nrf2 much exceed that of endogenous Nrf2, and that endogenous level of Keap1 is insufficient to regulate such excess of Nrf2. Consequently, the modification of endogenous Keap1 with sulforaphane would have little effect on the stability of overexpressed FT-Nrf2. In agreement with this conclusion, when FT-Nrf2 was co-expressed with unlabelled Keap1, we observed a time-dependent reduction in fluorescence lifetime of FT-Nrf2 upon sulforaphane treatment, indicative of Nrf2 stabilisation (Fig. 2d). Remarkably, this was apparent in both nuclear and cytoplasmic compartments, in agreement with a previous report showing that sulforaphane inhibits the ubiquitination of Nrf2 in both the cytoplasm and the nucleus^28^. We also observed that in the presence of Keap1, Nrf2 partially relocalised to the cytoplasm, consistent with ability of Keap1 to sequester Nrf2 into the cytoplasm by assisting nuclear export of Nrf2^29,30^.

**Fig. 3:**
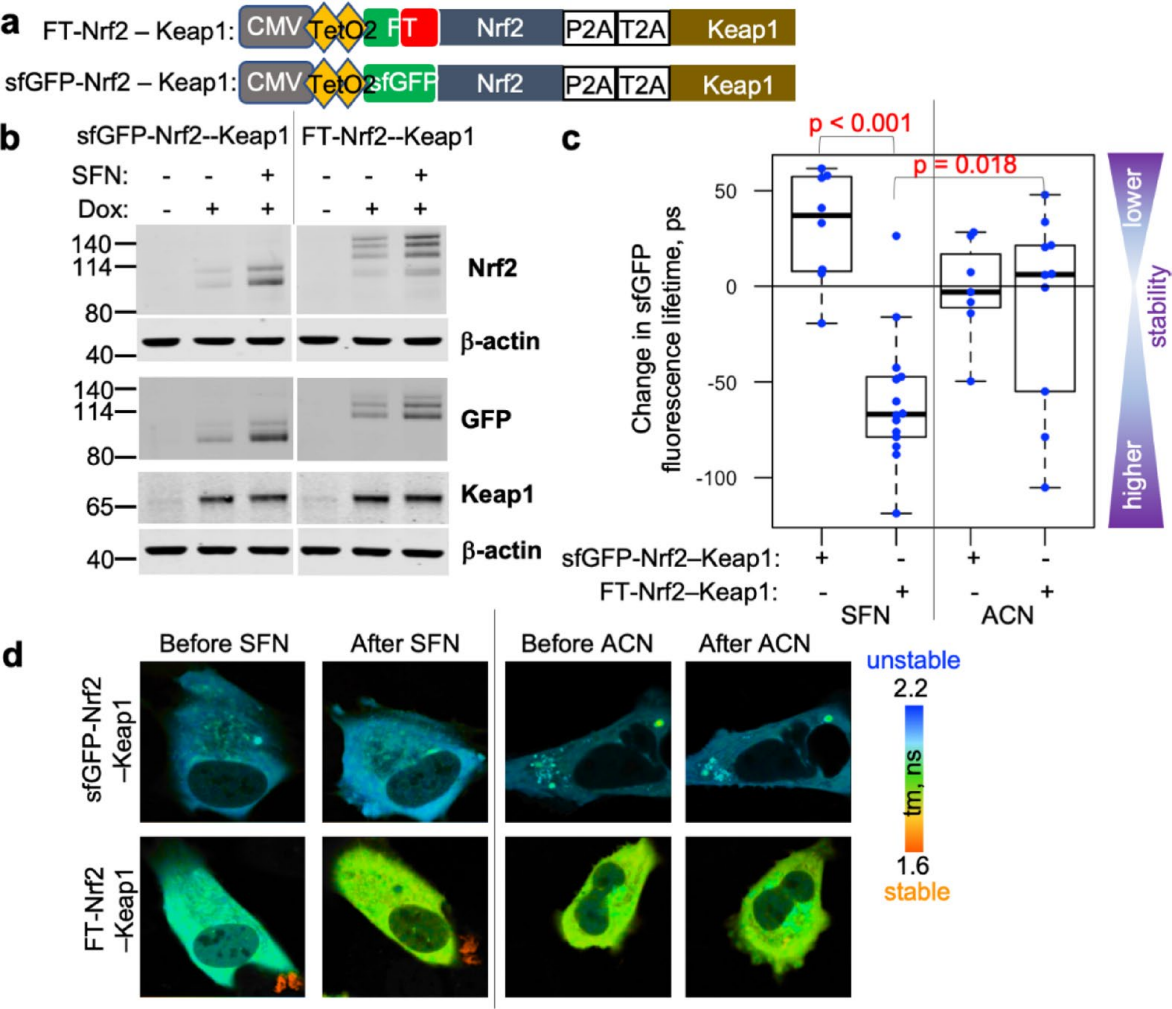
Measuring turnover of FLIM-Timer tagged Nrf2 stoichiometrically co-expressed with Keap1. (**a**) Bicistronic constructs for Dox-inducible co-expression of sfGFP-mCherry-Nrf2 (FT-Nrf2) or sfGFP-Nrf2 with unlabelled Keap1 at a constant ratio. sfGFP-mCherry (FLIM-timer) or sfGFP upstream of Nrf2 coding sequence is followed by two 2A peptides and a Keap1 coding sequence cloned into pcDNA5-FRT/TO vector (a part of Flp-In T-REx™ system) under vector’s CMV promoter controlled by two Dox-sensitive TetO2 elements. (**b**) U2OS-FRT/TO cells (Flp-In™ T-REx™ host U2OS cells with integrated FRT site and a Tet-repressor) with stably integrated FT-Nrf2-Keap1 or sfGFP-Nrf2-Keap1 constructs shown in **a** were either left untreated or induced with Dox for over 20 h. Dox-induced cells were further treated with 5 µM sulforaphane (SFN) for 3 h or left untreated. Cells were immunoblotted for Nrf2, GFP or Keap1, with β-actin as a loading control. Full blots are in Supplementary Information. (**c**) Fluorescence lifetime of FT-Nrf2-expressing U2OS-FRT/TO cells was significantly reduced upon SFN treatment, consistent with its stabilisation. U2OS-FRT/TO cells with stably integrated constructs shown in panel **a** were Dox-induced for 3 days and FLIM was measured before and after addition of either 5 µM SFN or equivalent volume of vehicle (acetonitrile, ACN) for 2 h. Each dot represents treatment-induced change in fluorescence lifetime in an individual cell (7–14 cells per group), corrected for any fluorescence intensity as described^9^. Median shown as thick lines, and boxes outline 75 percentiles of samples in Fig. 1d. (**d**) Representative fluorescence lifetimes (tm) colour maps of cells quantified in (**c**), before and after indicated treatment. Colour key is given on the right.

It was, however, difficult to quantify this effect due to a strong cell-to-cell variability and a lack of readout for the amount of ectopically-expressed Keap1 per cell. With the stoichiometry of inhibitory Keap1-Nrf2 complex of 2:1^31^, the degree of Keap1-dependent destabilisation and turnover of Nrf2 depends on their relative abundance. Transient co-expression of sfGFP-labelled Nrf2 and mCherry-labelled Keap1 that allowed visualisation of the relative levels of these two proteins in a similar system confirmed that it was highly variable among cells (Suppl. Figure 3). To reduce cell-to-cell variability in the levels and relative abundance of Nrf2 and Keap1, we generated a Dox-inducible bicistronic construct that combined FLIM-timer-Nrf2, or sfGFP-Nrf2 as a control, and an unlabelled Keap1, separated by two 2 A peptides shown to minimise read-through^32^ (Fig. 3a). This construct was integrated into a genomic FRT site within Flp-In™ T-REx™ host U2OS cells (U2OS-FRT/TO cells), giving rise to cells with Dox-inducible co-expression of FT-Nrf2 (or sfGFP-Nrf2 control) protein with Keap1 from a single promoter (Fig. 3b). Both sfGFP- and FLIM-timer-tagged Nrf2 were efficiently stabilised by sulforaphane treatment in this system (Fig. 3b). Importantly, the fluorescence lifetime of FT-labelled but not of sfGFP-labelled Nrf2 was statistically significantly reduced by sulforaphane (Fig. 3c, d), indicative of Nrf2 stabilisation, while vehicle (acetonitrile, ACN) had no effect on the fluorescence lifetime of FT-Nrf2. Thus, FT-Nrf2 stoichiometrically co-expressed with Keap1 can act as a sensitive reporter for the efficacy of Nrf2 inducers.

**Fig. 4:**
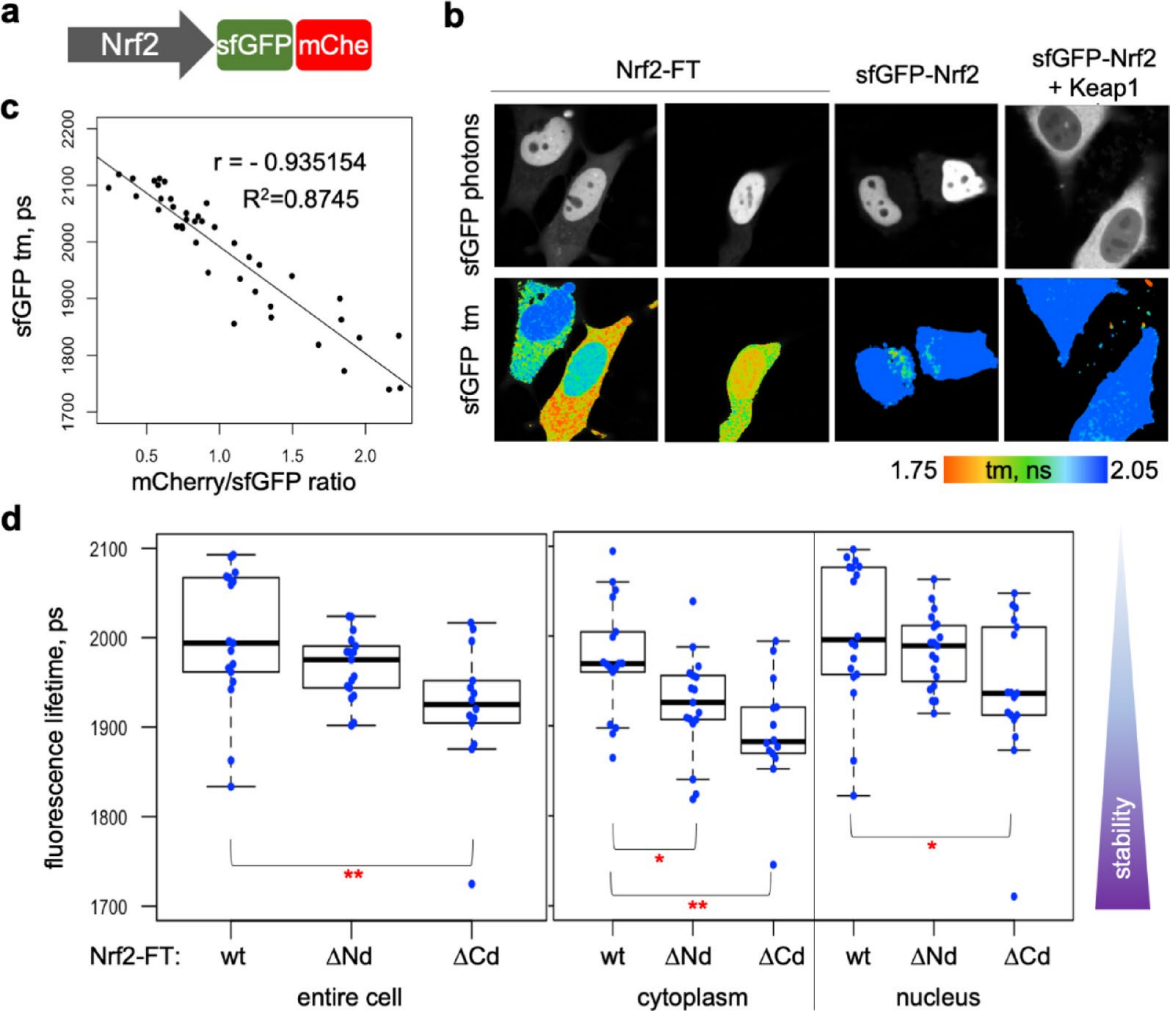
C-terminal FLIM-timer tagged Nrf2 as a readout of Nrf2 stability. (**a**) Schematics of Nrf2 labelled with sfGFP-based FLIM-timer at C-terminus. (**b**) Photon numbers (top) and fluorescence lifetime (tm, bottom) of HeLa cells transiently expressing Nrf2-FT(Nrf2-sfGFP-mCherry, left two panels) or donor-only control sfGFP-Nrf2 (with and without Keap1, right two panels), representative from 12, 3 or 5 FLIM measurements, respectively. The colour key for fluorescence lifetime is shown underneath. Note that Keap1-mediated destabilisation does not alter fluorescence lifetime of donor-only control. Quantifications are in Suppl. Fig 4. (**c**) Negative correlation between ratiometric and FLIM-based measurements in HeLa cells transiently transfected with Nrf2-sfGFP-mCherry with or without Keap1 within either cytoplasm or nucleus, pooled (N = 42). The ratio between mean mCherry and sfGFP fluorescence intensities with brightness above the threshold of 20 are plotted against globally binned fluorescence lifetime of sfGFP in the same region. Pearson Correlation Coefficient (r) and a goodness of fit (R2) are shown. (**d**) Fluorescence lifetimes of HeLa cells transfected with wild-type Nrf2 (wt, N=17 cells) or mutant Nrf2 lacking either both DLG and ETGE motifs of N-terminal Keap1-dependend degron (ΔNd, N =17 cells) or DSGIS motif serving as a C-terminal phospho-degron for β-TrCP-dependent degradation (ΔCd, N = 14 cells), tagged with FLIM-timer sfGFP-mCherry at its C-terminus, were measured using FLIM. Each dot represents mean fluorescence lifetime of entire cell (left) or cytoplasmic or nuclear region (right), and the boxplots show the data distribution as in Fig. 1d. Reduced fluorescence lifetime in Nrf2 mutants corresponds to a more stable protein. Significant differences are indicated by asterisks: *p<0.05, **p<0.01. See also Suppl. Fig. 4.

The Nrf2 stability is regulated primarily by Keap1-dependent cytoplasmic degradation via an N-terminal degron in Neh2 domain of Nrf2, and by SCF^β-TrCP^-dependent degradation in the nucleus via a phosphodegron within in Neh6 domain of Nrf2^33^. Consequently, N- and C-terminal tags might differentially affect accessibility and efficiency of the respective degrons. To assess the effect of tag position on Nrf2 turnover, we additionally constructed Nrf2 C-terminally tagged with FLIM-timer (Fig. 4a). Our previous study suggested that such construct is likely to be functional, since C-terminal tagging did not interfere with stabilisation of Nrf2 by the Keap1-targeting inducer CDDO^34^. In the absence of co-expressed Keap1, C-terminally tagged Nrf2-FLIM-timer still accumulated in the nucleus, but it was often (in 46% of cells) more stable (i.e. it had shorter fluorescence lifetime) in the cytoplasm than in the nucleus (Fig. 4b) compared to no such cells with N-terminally tagged Nrf2 (see Fig. 1e). The fluorescence lifetime of Nrf2-FT showed an excellent negative linear correlation with mCherry/GFP ratio (Fig. 4c), confirming that FLIM measurements reflected the maturation state of the fluorescence timer and provided a faithful measure of stability. Furthermore, the depletion of either a bipartite N-terminal degron for Keap1-Cul3 mediated degradation^35,36^ or a more C-terminal motif that recruits β-TrCP for SCF^β-TrCP^ -mediated degradation^10^ decreased fluorescence lifetime of Nrf2-FT (Fig. 4d), consistent with stabilisation of Nrf2 in the absence of these degrons. These results confirm the ability of C-terminal FLIM-timer to report on Nrf2 stability, even without Keap1 co-expression, and show that FT-timer can be used to assess differential effects of C- and N-terminal tagging on stability of fusion proteins.

### Monitoring mitotic exit by visualising degradation of Cyclin B1

**Fig. 5:**
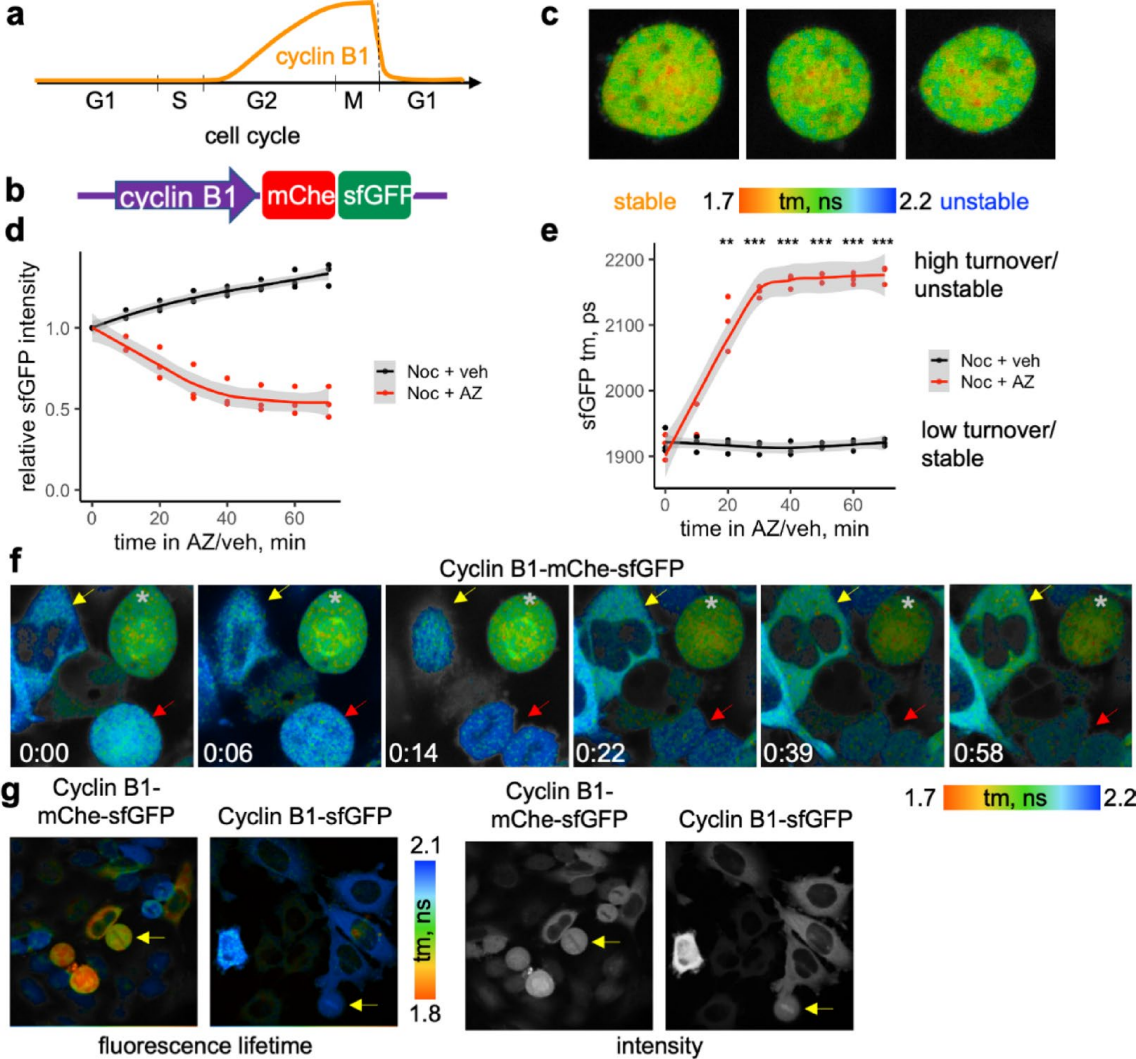
Monitoring mitotic exit with FLIM-timer-cyclin B. (**a**) Changes of cyclin B1 levels during cell cycle. (**b**) Cyclin B1 tagging strategy. Endogenous cyclin B1 in HeLa cells tagged with mCherry-sfGFP, with linker shown in Fig 1b. (**c**) Examples of fluorescence lifetime (tm) in nocodazole-arrested cells expressing cyclin B1-mCherry-sfGFP. The tm values color-coding shown underneath. Tm here and below was measured using InTune laser at 490 nm excitation. (**d**,**e**) HeLa cells expressing cyclin B1-mCherry-sfGFP were treated for 1 h with Nocodazole prior addition of 1.25 μM AZ 3146 or equal volume of DMSO (veh) for 70 min. Mitotic (rounded) cells were followed by a time-lapse FLIM and normalised sfGFP fluorescence intensity (**d**) or the mean fluorescence lifetime (**e**) in each cell were plotted against time of treatment. Dots are individual cell values and lines are fitted local polynomial regression curves with the 95% confidence interval shown as grey shading. Statistically significant (**) and highly statistically significant (***) differences are indicated above. (**f**) Time course FLIM of unsynchronised culture of HeLa cells expressing cyclin B1-mCherry-sfGFP, with time from the start of the imaging (hour:min). The color-coding for fluorescence lifetimes is shown underneath. A cell that remains in mitosis throughout the time course is marked with asterisk; a red arrow points to a cell that exits mitosis and divides; and the yellow arrow shows to a large interphase cell that reduces fluorescence lifetime and increases its intensity over the time course, consistent with G2 stage of cell cycle. (**g**) Reduced fluorescence lifetime is not observed in mitotic cells overexpressing donor-only control, Cyclin B1-sfGFP. FLIM was acquired in unsynchronised culture of HeLa cells expressing either cyclin B1-mCherry-sfGFP (left) or cyclin B1-sfGFP(right). Tm maps are on the left two panels with color-coding shown on the right, and the sfGFP intensity (photon number maps) are on the right two panels. Note that mitotic cells (yellow arrows) display low fluorescence lifetime only in the Cyclin B1-mCherry-sfGFP expressing and not in Cyclin B1-sfGFP expressing sample.

We next asked whether FLIM-timer can be also employed to monitor cell cycle transitions, by visualising degradation of cyclins that drive cell cycle progression. Cyclin B1 gradually accumulates during G2 stage of cell cycle reaching its highest level in mitosis (Fig. 5a). Cyclin B1 continues to be transcribed throughout M-phase^37^, while its degradation in early mitosis is inhibited by the so-called spindle assembly checkpoint (SAC) until all chromosomes are properly attached to the mitotic spindle. Mechanistically, an active SAC prevents the Anaphase-Promoting Complex/Cyclosome (APC/C), an E3 ubiquitin ligase, from targeting cyclin B for degradation^38^. When attachment of all chromosomes is completed, satisfaction of the SAC causes an abrupt degradation of cyclin B1 by APC/C in a complex with its co-activator CDC20 that triggers mitotic exit (Fig. 5a). The timing and kinetics of cyclin B1 degradation is of crucial importance for understanding the regulation of cell division, karyotype stability and the efficiency of mitosis-targeting anti-cancer drugs.

We wondered whether the FLIM-timer approach could simplify the assessment of cyclin B1 degradation. In order to avoid any potential artefacts associated with overexpression, we used CRISPR-Cas9-mediated insertion to tag the endogenous cyclin B1 with FLIM-timer. Several additional modifications were used in the design of these constructs. First, we decided to tag cyclin B1 with FLIM-timer on the C-terminus, as previous studies had established that a C-terminal tagging of cyclin B1 does not interfere with mitotic progression or cyclin B1 degradation^39–41^. Second, following the recommendations for the design of C-terminal tFT^25^, we inverted the positions of the fluorophores (Fig. 5b). Finally, we removed the first methionine from all fluorophore-coding sequences to reduce potential translation initiation from these sites. Cells expressing such endogenously tagged Cyclin B1 had the same proliferation rates as parental HeLa cells, indicating that the constructs were not hazardous.

HeLa cells with mCherry-sfGFP-tagged cyclin B1 were synchronised in mitosis using nocodazole that destabilises microtubules preventing cells that enter M-phase from forming mitotic spindles (Fig. 5c-e). This arrests cells with high level of cyclin B1 and an active spindle checkpoint^40,42^. The fluorescence lifetime of mitotically arrested cells was shortened to 1918.76 ± 17.29 ps (compared with 2176.20 ± 24.11 ps of sfGFP alone, see Fig. 1d), indicating that cyclin B1-FT protein is stable in these cells (Fig. 5c). We then treated such cells with AZ 3146, a selective inhibitor of a SAC-regulating kinase Mps1^43^. We and others have previously shown that such treatment overrides SAC and causes cyclin B1 degradation and mitotic exit in the presence of nocazadole^42^. AZ 3146 triggered a clear decline in the level of fluorescent cyclin B1 in nocodazole-arrested cells (Fig. 5d, red), confirming that AZ 3146 induced degradation of cyclin B1. Mitotic exit was confirmed by changes in cell morphology by the end of the time course. The sfGFP fluorescence lifetime of cyclin B1-mCherry-sfGFP had started to increase within 10 min of AZ 3146 addition and reached maximal values close to that of unquenched sfGFP, indicative of fully destabilised protein, at about 30 min of treatment (Fig. 5e, red), while vehicle treatment did not cause any change in the fluorescence lifetime of nocodazole-arrested cells (Fig. 5e, black).

The intensity of endogenous cyclin B1-mCherry-sfGFP in interphase cells was sufficient to observe changes in cyclin B1 turnover throughout the cell cycle. Repeated FLIM measurements of untreated unsynchronous population of HeLa cells expressing this construct (Fig. 5f) allowed not only to detect the stabilised cyclin B1 in mitotic cells (asterisks), but also to observe destabilisation of cyclin B1 upon mitotic exit and cell division (red arrows) and a gradual decrease in the turnover of cyclin B1 in some of interphase cells that are presumably at G2 stage of cell cycle, apparent as a decrease in fluorescence lifetime (yellow arrow). This was not observed in cells expressing donor-only tagged CyclinB1 (Fig. 5g).

Thus, sfGFP-based FLIM-timer is suitable to monitor cyclin B1 degradation during mitotic exit, confirming the general applicability of this approach.

## Discussion

We present here a novel concept to use FRET as readout of fluorescent timer for measuring protein stability or turnover. Fluorescent timer applications utilise kinetics of their maturation apparent from changes in either their spectra (for single-molecule fluorescence timers) or intensity (for tandem fluorescent timers that are composed of two fluorophores with different maturation rates). Using the regulatable degradation of Nrf2 and mitotic degradation of cyclin B as models, we here show that the ability of mCherry to act as a FRET-acceptor also develop with time of its maturation, allowing the use of FLIM-FRET as a readout of the fluorescent timer. FRET acceptor causes a non-radiative efflux of energy from a proximal donor fluorophore, on condition that the absorption spectrum of the FRET acceptor overlaps with the emission spectrum of the FRET donor, and their respective emission and absorption dipoles are not perpendicular^18^. For the mCherry used in this study, the maturation process proceeds through formation of several intermediate molecules with spectral properties distinct from those of mature mCherry with its characteristic stable excitation and emission peaks at 587 nm and 610 nm, respectively. The fluorophore maturation starts from autocatalytic cyclization of chromophore-forming tripeptide within β-barrel, followed in most GFP-like fluorophores by the rate-limiting dehydration and oxidation steps^22,44^. In red-shifted fluorophores, additional steps are required for full maturation, though the exact mechanism differs between fluorophores^45^. A branching point at dehydration step was suggested to direct the fluorophore maturation toward formation of red fluorophores, via a blue intermediate^46^. An alternative mechanism of red fluorophore maturation proceeds via a green fluorescent state^45^and the existence of such green intermediates have been shown for DsRed and its derivative mCherry^47,48^. Whether these intermediate states have appropriate absorption spectrum with sufficient extinction coefficient to mediate FRET from a green fluorophore such as sfGFP has not been explored. While it is clear that the absorbance spectra of blue intermediates should not overlap with the emission of a green FRET donor, the potential of green or colourless intermediates to serve as FRET acceptors is not known. The lack of acceptor fluorescence emission does not necessarily preclude FRET accepting, as apparent from the existence of dark FRET acceptors^49,50^. Furthermore, FRET between fluorophores of identical spectra (e.g. homo-FRET) is also possible^51^. However, the tight correlation between relative intensity of mCherry within FLIM-timer and its ability to mediate FLIM-FRET that we observed here (Figs. 1f and 4c) suggests that the maturation intermediates of mCherry have only minimal contribution to mediating FRET from the green FRET donor, and that ability to accept FRET is the property of the fully mature mCherry.

The principal advantage of the method described here is that it utilises fluorescence lifetime for measuring protein turnover with fluorescent timers, which in turn makes it independent on fluorescence intensity and allows comparison between bright and dim cellular compartments. It is particularly useful for strongly compartmentalised target proteins such as Nrf2. The mechanism of Nrf2 stabilisation by Nrf2 inducers is not completely understood, and one of the existing models suggests that inducer-modified Keap1 traps Nrf2 in the cytoplasm within dysfunctional Cullin3-Keap1 E3 ligase complex, before newly synthesised Nrf2 moves to the nucleus^52^. The newly developed FLIM-timer technology made it possible for the first time to experimentally test this hypothesis in live cells. In support of the above model, our data revealed that the electrophile sulforaphane causes Keap1-dependent cytoplasmic stabilisation of Nrf2 (Fig. 2d). Our approach also confirmed the contribution of Keap1-independent degradation pathways, such as SCF^β-TrCP^, to the basal Nrf2 turnover (Fig. 4d).

Throughout this work, the fluorescence lifetime data were analysed using a single-exponential decay model. This model is a simplification, since the fluorescence lifetime of FLIM-timer in each pixel or cellular area is likely to be a combination of fluorescence lifetimes of unquenched FRET donor and FRET donor quenched to a different degree at multiple stages of acceptor maturation, - thus, it would be best described by a multiexponential or stretch-exponential decay model^53^. The presence of more than two components is indeed apparent from the partial mismatch of the 2-component model and the photon trace shown in the Suppl. Figure 2a. However, the simplified analysis with one-component exponential model was only marginally less fitting (compare R^2^ for both models in Suppl Fig. 2a), showed an excellent correlation with the previously used protein stability measurements (Fig. 1f), and required much fewer photon data than the more complex analytical models, allowing to perform FLIM acquisition at lower laser illumination intensity or for a shorter time, reducing photobleaching and loss of resolution due to cell movement. We have previously shown the validity of such simplification, confirming that analysing the 2-component FLIM with single-exponential fitting produces very similar pattern of fluorescence lifetimes^19^.

In conclusion, we show that the FLIM-timer-tagged Nrf2 is a useful tool for monitoring and localising changes in Nrf2 stability, and that the same approach can be applied to monitor cyclin B1 degradation during mitotic exit. The method described here greatly increases the applicability of fluorescent timers for visualisation and quantification of protein turnover, expanding it to samples with complex expression pattern of the protein of interest not suitable for classical Fluorescent Timer measurements, and thus has a potential to provide new biological insights.

## Materials and methods

### Reagents

Doxycycline hyclate (Dox, Sigma/Merk, D9891), *R*,* S*-sulforaphane (LKT Labs, S8044), nocodazole (EMD Millipore, 487928), AZ3146 (Selleckchem, S2731). Antibodies for immunoblotting: rat monoclonal anti-Keap1, clone 144 (Millipore, MABS514, 1:10000 dilution); rabbit anti-Nrf2 rb (Cell Signalling, D1Z9C, cat Nr 12721P, 1:1000 dilution); rabbit polyclonal anti-EGFP (AbCam, ab6556, 1:1000 dilution) that detects sfGFP; anti-mCherry (Living Colors, 1:1000 dilution), mouse monoclonal anti-β-actin, clone AC-15 (Sigma, A 5441, 1:20000 dilution); anti-rabbit, anti-rat and anti-mouse IRDye800 or IRDye-680 labelled secondary antibodies (Li-COR Biosciences, 1:15000 dilution).

### Plasmids

Plasmids encoding an unlabelled mouse Keap1 and fluorescently labelled mouse sfGFP-Nrf2 and mouse Keap1-mCherry (Keap1-12fl-mCherry) were described previously^19,54^. pmCherry-N1 and pEGFP-C1 are from Clontech, sfGFP-C1 (N54579) is from Addgene, and pcDNA5-FRT/TO and pOG44 are from ThermoFisher. The construct for transient overexpression of FT-Nrf2 (sfGFP-mCherry-Nrf2) was cloned by inserting a fragment comprising a linker followed by a mCherry coding sequence excised with BsrGI from a previously described pEGFP-mCherry plasmid^3^ into the BsrGI site of sfGFP-Nrf2. The plasmid for transient Nrf2-FT expression (Nrf2-sfGFP-mCherry, DU58475) was constructed by replacing the first half of mCherry sequence between AgeI and BbvCI restriction sites in a previously described Nrf2-mCherry^34^ (DU58450) by the AgeI-BbvCI fragment from the above FT-Nrf2 plasmid that encompassed sfGFP, linker and the first half of mCherry. The mutant Nrf2-FT lacking C-terminal bi-partite degron for Keap1-mediated degradation (ΔNdNrf2-FT, DU58523) was generated from Nrf2-FT by sequential QuickChange mutagenesis that replaced nucleotides 76[caagacatagatcttgg]92 of Nrf2 ORF by 76[TCagacatagCtGGtgA]92 and removed nucleotides 235[gaaacaggagaa]246, thereby producing the Nrf2 protein carrying S26Q, D29A, L30G and G31E substitutions and 79[ETGE]82 depletion. The mutant Nrf2-FT lacking a more C-terminal GSK3β-dependent degron (ΔCdNrf2-FT, DU58532) was generated by deleting nucleotides 1000[gactctggcatttca]1014 of Nrf2 ORF, resulting in removal of the amino acids 334[DSGIS]338. sfGFP-Nrf2-mCherry (DU58516) was constructed by subcloning the HindIII/MfeI insert from mNrf2-mCherry containing the C-terminal portion of mNrf2 and the mCherry open reading frame into the same sites of sfGFP-Nrf2. pcDNA5D FRT/TO sfGFP (DU58501) and pcDNA5D FRT/TO sfGFP-mCherry (DU58502) were made by PCR amplifying the sfGFP or sfGFP-linker-mCherry sequences from above Nrf2-sfGFP-mCherry (DU58475) and cloning it as a BamHI/NotI insert into the same sites of pcDNA5D FRT/TO (pcDNA5 FRT/TO (ThermoFisher) with modified multiple cloning sites, MRC-PPU reagents and services, DU41459). pcDNA5D-FRT/TO-sfGFP-AID (DU58517) and pcDNA5D-FRT/TO-sfGFP-mCherry-AID (DU58520) were made by PCR amplifying a linker-flanked AID sequence from pAID1.1-N (kind gift from Kevin Hiom) and cloning it as a NotI-NotI insert into the NotI site of above pcDNA5D FRT/TO sfGFP (DU58501) or pcDNA5D FRT/TO sfGFP-mCherry (DU58502). Plasmid for bicistronic co-expression of sfGFP-mCherry-Nrf2 with unlabelled Keap1 (pcDNA5-FRT/TO-sfGFP-mCherry-Nrf2–Keap1) was produced from pcDNA5D-FRT/TO-sfGFP-mCherry-AID by replacing “mCherry-AID” fragment by an insert comprised of mCherry-Nrf2, followed by P2A and T2A peptide sequences from pGEMT-PTE2A (kind gift of Li Qian) and followed by Keap1, in a sequential multi-step cloning. For the pcDNA5-FRT/TO-sfGFP-Nrf2–Keap1 plasmid served as a control, mCherry sequence was not included into an insert. All constructs were sequence-validated.

The construct for endogenous tagging of Cyclin B1 with FLIM-timer was produced by Gibson assembly of the AAV_cyclin B-EYFP vector^39^ with the GeneArt custom DNA fragment (ThermoFisher Scientific) containing the mCherry ORF, SGLRSRA linker, followed by sfGFP ORF (both fluorophore sequences without first methionine-coding triplet), flanked by the 57–60 nucleotide overhang sequences that matched cyclin B-LHA and cyclin B-RHA from the AAV cyclin B-EYFP modified to include BspEI, SalI and NotI restriction sites. Cyclin B1-sfGFP plasmid was made by excising mCherry from cyclin B1-mCherry-sfGFP using BspEI sites around mCherry ORF. All constructs were sequence-validated.

### Cell lines

HEK293 and HeLa cells were cultured in DMEM media containing 10% heat-inactivated Fetal Bovine Serum. Flp-In™ T-REx™ host cells U2OS-FRT-TO (kind gift of Dr Laureano de la Vega) and HeLa-FRT-TO (kind gift of Prof Stephen Taylor) were maintained in DMEM media containing 10% heat-inactivated Fetal Bovine Serum. For integration of Dox-inducible clones into Flp-In™ T-REx™ host cells, cells were co-transfected with 1:1 mixture of the construct of interest cloned into pcDNA5-FRT-TO vector and pOG44 plasmid encoding Flip recombinase, using Lipofectamine 2000 (Invitrogen). The cells with successful integration were selected under 250 µg/ml hygromycin B (ThermoFisher Scientific).

Endogenous cyclin B1 was tagged using CRISPR-Cas9 knock-in strategy. HeLa-FRT-TO cells were transfected with homology arms (CyclinB-mCherry-sfGFP or CyclinB-sfGFP), gRNA (TGTAACTTGTAAACTTGAGT) and pcDNA5-Cas9-WT-NLS, using FuGENE HD (Promega) according to manufacturer’s protocol. 5 h after transfection, cells were treated with 1 µg/mL doxycycline (Sigma) for 3 days to trigger Cas9 expression. Tagged cyclin B1 cells were then enriched by FACS sorting. Sorted cells were collected and cultured for 1 week in collection media – DMEM (Gibco) supplemented with 20% FBS (Gibco), 50 µg/mL penicillin/streptomycin (Thermo Fisher), 100 µg/mL Normocin™ (InvivoGen) and 30% conditioned media (filtered from cells after 2–3 days culture).

### Cell transfections and treatments

Transient transfections of cell lines were performed using calcium phosphate method as described^9^. Prior imaging, cell culture media was replaced by phenol-free DMEM with all required supplements specific to cell line. To induce expression of integrated constructs in generated cells with Dox-inducible integrated constructs, 0.5–1 µg/ml Doxycycline hyclate (Dox, Sigma/Merk) was added to the media for more than 20 h. Treatments added during imaging were pre-diluted in 20–100 µl of imaging media at the concentration required to achieve indicated final concentration in the sample. The same dilution of vehicle was used as a control.

### Immunoblotting

Cells grown on 6-well plates were washed twice with PBS and lysed in SDS lysis buffer containing 2% SDS, 62.5 mM TrisHCl pH 6.8, 10% glycerol and protease inhibitors (cOmplete Mini, EDTA-free protease inhibitor cocktail, Roche), boiled for 2 min, sonicated and supplemented with DTT up to 100 µM final concentration and small amount of Bromophenol Blue. 10 µg of lysates were separated on 4–12% pre-cast BisTris NuPAGE gels (Invitrogen) under MOPS running buffer alongside a PageRuler™ protein ladder (ThermoFisher Scientific, 26616) and transferred onto Amersham™ Protran^®^ Premium 0.45 μm Nitrocellulose membrane under transfer buffer containing 25 mM Tris, 129 mM Glycine and 20% Methanol. Membranes were stained with 1% Ponceau S in 5% acetic acid to confirm equal transfer, de-stained and blocked in 5% non-fat milk in PBST (PBS supplemented with 0.1% Tween 20) for 1 h before blotting with primary antibodies diluted to indicated concentration in above blocking solution overnight at 4 °C. Membranes were washed in PBST, incubated with appropriate secondary antibodies diluted 1:15000 in blocking solution for 1 h at room temperature, washed again and scanned using Odyssey CLx Infrared Imaging System. Alternatively, blots were incubated with 1:5000 diluted HRP-labelled secondary antibody followed by development with ECL reagents (Amersham) and exposure to film. Quantifications were performed either in Image Studio Lite (Version 5.5.4, LiCOR) using Western analysis tool with lane background, or in Fiji, by quantifying the band intensity after subtracting the background intensity. All values were first normalised to actin level in the same samples, and subsequently normalised to indicated reference sample.

### Imaging

Life cells were imaged on LSM 710 (Zeiss) confocal microscope operated by ZEN software (Zeiss) equipped with dark environmental chamber maintained at 37 °C and a humidified source of 5% CO_2_. Cells growing on glass-bottom dishes (35 mm FluoroDishes, World Precision Instruments, or 35 mm 4-compartment CELLview dishes, Greiner Bio-One) were held in heated Petri-dish holder insert and imaged with 488 nm (for EGFP or sfGFP) or 594 nm (for mCherry) confocal lasers using 63x/1.4NA oil immersion objective. Within each experiment, the laser settings and acquisition parameters were identical between samples. Images were processed to adjust displayed intensities, generate maximal intensity projections, add annotations and create movies in ImageJ/FIJI or OMERO.

### FLIM acquisition and analysis

Fluorescence lifetime was measured using SPC-150 Time-Correlated Single Photon Counter (TCSPC) module (Becker&Hickl) attached to LSM 710 microscope (Zeiss) equipped with either InTune pulsed laser with repetition rate 40 MHz and tunable wavelength (Zeiss) or 2-photon Chameleon laser with pulse repetition rate 80 MHz (Coherent) and HPM-100-40GaAsP detector that feeds to TCSPC module. FLIM data acquisition is operated by SPCM software (Becker&Hickl) controlling TCSPC module. sfGFP fluorescence lifetime was measured at 490 nm (InTune laser) or 920 nm (2-photon laser) wavelength and the 500–550 nm bandpass filters suitable for GFP. For 2-photon FLIM, additional 690 nm short-pass filter was used to cut off far-red and infrared light. Each measurement was acquired for 40–120 s with laser intensities adjusted to achieve Constant Fraction Discriminator (CFD) rate between 5 × 10^5^ and 1 × 10^6^ events per second, with time resolution of 256 time-channels and spatial resolution of 512 × 512 or 256 × 256 pixels. The duration of acquisition and resolution were kept constant among all samples of the same experiment. FLIM generated the cumulative photon traces within each pixel of the image, which can be used to determine fluorescence lifetime(s) by fitting an appropriate exponential decay model (generic formula 1 or 2 below).

1 $$\:\text{O}\text{n}\text{e}-\text{e}\text{x}\text{p}\text{o}\text{n}\text{e}\text{n}\text{t}\text{i}\text{a}\text{l}\:\text{d}\text{e}\text{c}\text{a}\text{y}\:\text{m}\text{o}\text{d}\text{e}\text{l}:\:{F\left(t\right)=ae}^{-t/\tau}$$

2 $$\:\text{T}\text{w}\text{o}-\text{e}\text{x}\text{p}\text{o}\text{n}\text{e}\text{n}\text{t}\text{i}\text{a}\text{l}\:\text{d}\text{e}\text{c}\text{a}\text{y}\:\text{m}\text{o}\text{d}\text{e}\text{l}:F\left(t\right)=\:{a1e}^{-t/\tau1}+{a2e}^{-t/\tau2}$$

where τ, τ1 and τ2 are fluorescence lifetime components, $\:a1$ and $\:a2$ are the relative amplitudes of each component with $\:a1+a2=100\%$, and *t* is an independent variable.

This analysis was performed within SPCImage (Becker&Hickl), where fluorescence lifetime was determined by fitting 1-component (in the majority of analysis) or 2-component (where indicated) exponential decay models adjusted to remove the signal from Instrument Response Function (IRF) by deconvolution to the binned data^55^. For the fluorescence lifetimes datasets that included values exceeding 1/5th of the inter-pulse period (such as mNeonGreen constructs measured using 2-photon FLIM), we used the “incomplete decay” algorithm that also accounts for the residual signal from the previous pulse decay (the details could be found in the bh TCPSC handbook^55^. For the pixel-by-pixel analysis (used for generating images or calculating mean fluorescence lifetimes and changes in fluorescence lifetime as indicated), bin 3 or 4 was used. Alternatively, global binning was used as previously described^19^ by pulling together the data from all pixels within an outlined region of interest (nucleus, cytoplasm or entire cell).

For 2-component analysis, the second component was fixed at the average value obtained by measuring the FRET donor-only controls, and the amplitude weighted average fluorescence lifetime was calculated within the SPCImage software following formula 3:

3 $$\:\text{t}\text{m}\:=\frac{a1\times\tau1+\:a2\times\tau2}{a1+a2}$$

FRET efficiency (E) was calculated from the 2-component exponential fitting as

4 $$\:E=1-\frac{tm}{\tau2}$$

where tm is amplitude weighted average fluorescence lifetime and τ2 is the longer fluorescence lifetime component corresponding to fluorescence lifetime of FRET-donor only control.

Alternatively, the binned photon traces were exported from SPCImage and further analysed in R, where the 1- or 2-component exponential decay models were fitted to the same dataset after removing the initial portion of the curve containing IRF, plotted together and compared using ANOVA. Goodness of fit for each model was calculated in R as (5)

5 $$\:1-RSS/TSS$$

where *RSS* is a residual sum of squares, and *TSS* is total sum of squares.

The correlations between sfGFP fluorescence lifetime or FRET efficiency and the mCherry/GFP ratio were performed in R using fluorescence lifetimes values obtained with fitting the indicated model to the globally binned (pooled) FLIM data from nuclear or cytoplasmic cellular regions, and the mCherry/GFP ratio values calculated in OMERO within the same regions of the cells re-imaged by confocal microscopy in the instrument that permits sequential FLIM and confocal imaging of the same specimen.

For calculating CV in each type of measurement, sequential FLIM and confocal images of the same cells were analysed using pixel-by-pixel approach. The data files containing either fluorescence lifetimes obtained by 1-component fitting in each pixel of the image within outlined cellular area, or the number of photons (measure of GFP intensity) detected in the same pixels were exported from SPCImage and combined into matrix. Similarly, a matrix containing the mCherry/GFP intensity ratio and GFP intensity in each pixel of the same cellular area was constructed from the confocal image of the same cells. Both matrices were analysed in R, whereby pixels of similar GFP intensities were grouped into 37 bins of equal width, and the CV for fluorescence lifetime or mCherry/GFP ratio for pixels within each bin was calculated as CV = standard deviation/mean x 100. The obtained values were plotted against the middle intensity of each bin, normalised to the average GFP intensity of the entire cellular area. Time-lapse FLIM was performed as previously described^9^. To calculate intensity-independent changes in fluorescence lifetime or analyse FLIM time-lapses, the data from SPCImage were exported, processed in ImageJ and analysed within FLIMDAST (https://github.com/DinaDikovskaya/FLIMDAST) following a previously described pipeline^9^. Within each experiment, all FLIM data/images were acquired using the same optical settings and processed identically (including magnification factor). The intensity images represent the map of photon numbers acquired in each pixel.

### Data analysis

Data were assembled in Excel and analysed and plotted in R. Goodness of fit (R^2^, Pearson correlation coefficient (r) and p-values were calculated in R. P-values were determined using ANOVA with post-hoc Tukey HSD test (Fig. 4 and Suppl Fig. 2a) or Welch two sample two-tailed t-test (Fig. 5 and Suppl. Figure 2b).

## Supplementary Information

Below is the link to the electronic supplementary material.

Supplementary Material 1

## Data Availability

The original images and FLIM data files were deposited into BioImage Archive (DOI: 10.6019/S-BIAD1305) and can be accessed using the following link: https://tinyurl.com/FLIMtimerData.
