# Supplementary material for: A fluorescence lifetime-based FLIM-timer for measuring the protein turnover of transcription factor Nrf2 in live cells
Source: Sci Rep. 2025 Aug 14;15:29772. doi: 10.1038/s41598-025-14721-6 (PMC12350768; doi:10.1038/s41598-025-14721-6)
Supplement: Supplementary file 1 — Supplementary Material 1 [file 41598_2025_14721_MOESM1_ESM.pdf]

Supplementary Information

A Fluorescence Lifetime-Based FLIM-Timer for Measuring the Protein Turnover of Transcription Factor Nrf2 in Live Cells

Dina Dikovskaya[1,2,3*], Claudia Bento-Pereira[1], Kanade Shiga[1,4], Andrea Corno[1], Maureen Higgins[1], Rachel Toth[5], Adrian T. Saurin[1] and Albena T. Dinkova-Kostova[1,6,7]

[1]Division of Cellular Medicine, University of Dundee School of Medicine, Dundee, DD1 9SY, United Kingdom
[2]MRC Protein Phosphorylation and Ubiquitylation Unit, University of Dundee, Dundee DD1 5EH, United Kingdom
[3] Peninsula Medical School, Faculty of Health, University of Plymouth, Plymouth, PL4 8AA, United Kingdom
[4]The Department of Advanced Health Science, Graduate School of Advanced Health Sciences, Saga University
[5]MRC Reagents and Services, University of Dundee, Dundee, DD1 5EH, United Kingdom
[6]Department of Physiology, Pharmacology and Therapeutics and Department of Medicine, Johns Hopkins University School of Medicine, Baltimore, MD, USA
[7]Department of Medicine, Johns Hopkins University School of Medicine, Baltimore, MD, USA

[*]corresponding author

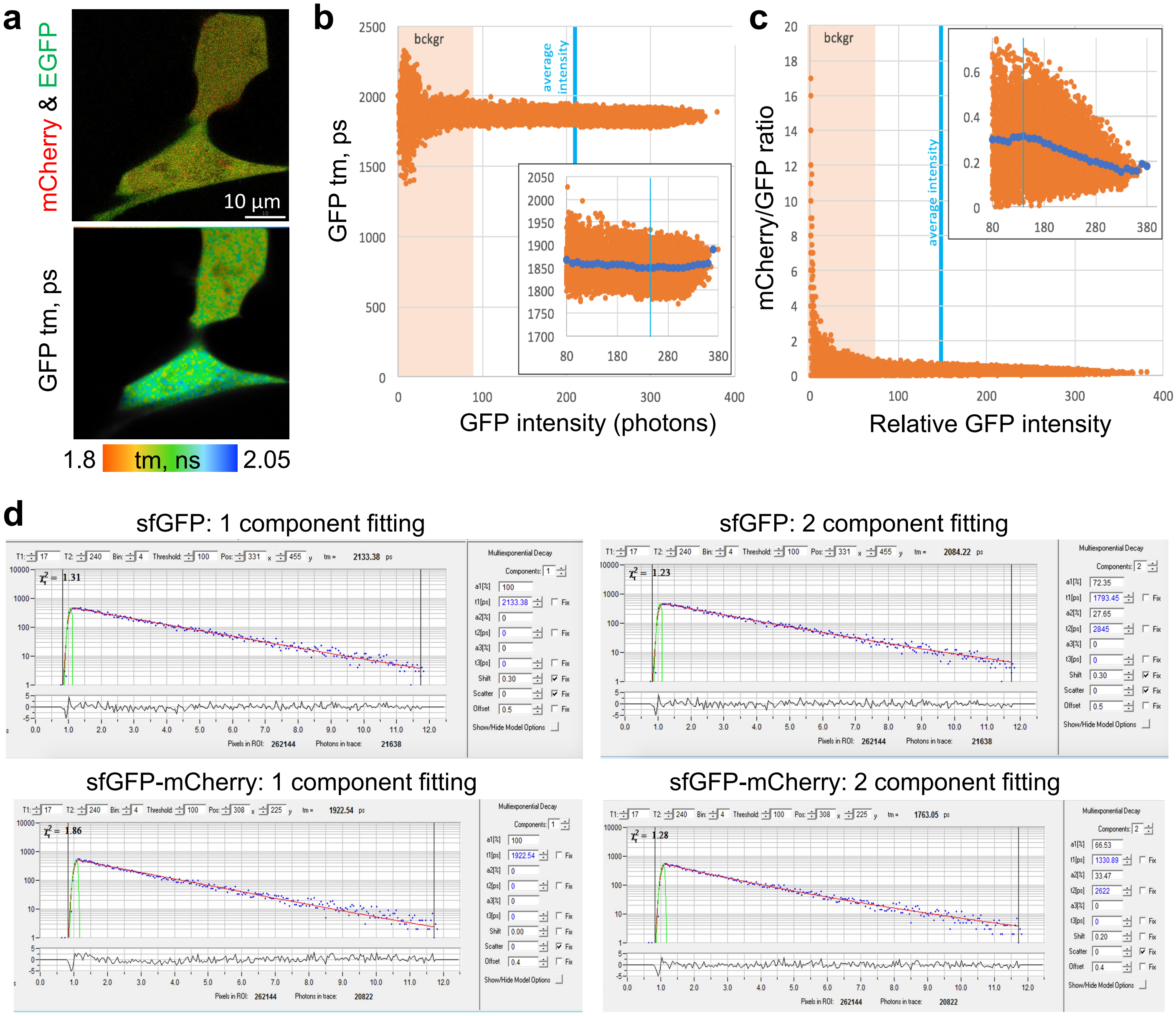

Supplementary Figure 1. Related to **Figure 1. (a)** HEK293 cells transfected with EGFP-mCherry imaged with confocal laser in GFP and mCherry channels (top panel, mCherry in red and EGFP in green) followed by FLIM with InTune laser (bottom panel, GFP tm color-coded as shown underneath). **(b, c)** Fluorescence lifetime (tm) values (**b**), or mCherry/GFP intensity ratios (**c**) plotted against photon number/fluorescence intensity in the same pixels of cells shown in **a**. Background fluorescence intensities are shaded, and average cellular intensity shown as light-blue vertical lines. The inserts zoom into average values above background. Dark blue plots in insets are average values of tm (**b**) or mCherry/GFP ratios (**c**) calculated for the 10-unit bins across the range of intensities. **(d)** Analysis of fluorescence lifetime in cells transfected with indicated constructs described in Figure 1d, performed using either 1-component (left) or 2-component (right) exponential fitting within SPCImage software. The selected analytical model (red line) is fitted to the measurements (blue dots) representing the number of photons (y-axis) detected at a particular time after the excitation pulse (plotted on x-axis, measured in nsec), within set time range (vertical lines). χ^2 at the top left indicates the goodness of fit, and residuals are shown underneath the main panels. The green curve is the Instrument Response Function estimated automatically from the shape of the rising edge of the data curve. The number of components within the fitted exponential model is shown at the top of "Multiexponential Decay" panels at the right, which also contains other parameters used in the fitting. The curves are for representative pixels of binned images, with bin = 4. The fitted fluorescence lifetime value (tm) is shown at the bar above each main panel.

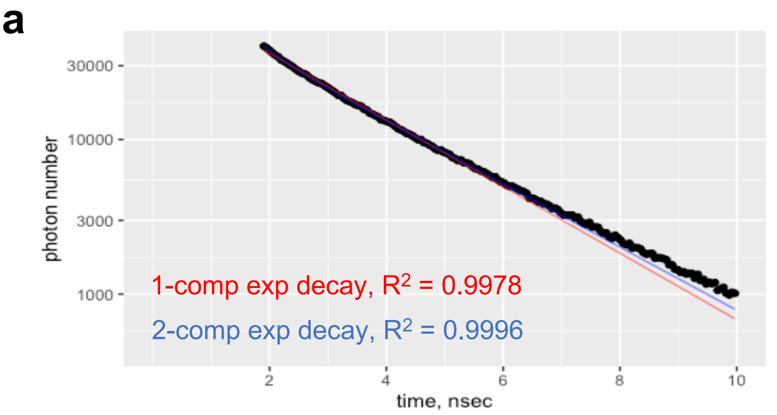

model	τ	τ1	τ2 (fixed)	$a1$	$a2$
1-comp	2.015				
2-comp		0.3883	2.1287	0.1148	0.8852

model	R^2	Pr(>F)
1-comp	0.9978	
2-comp	0.9996	<0.001

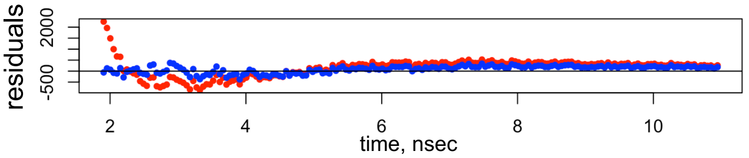

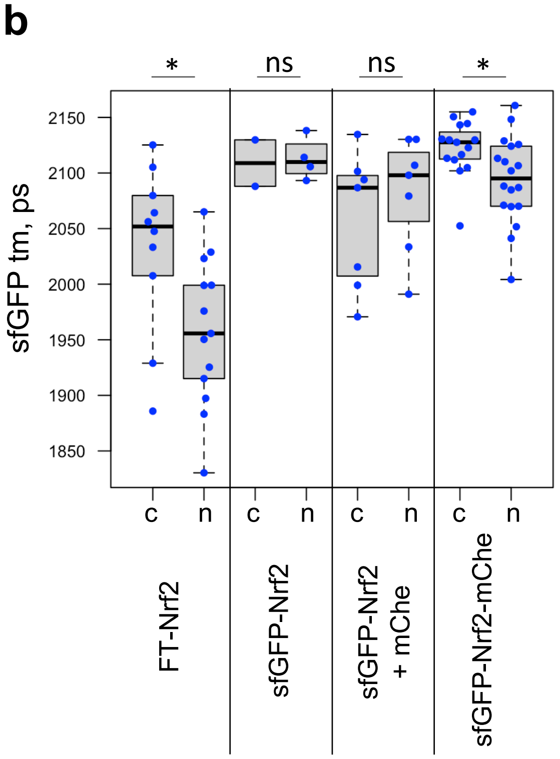

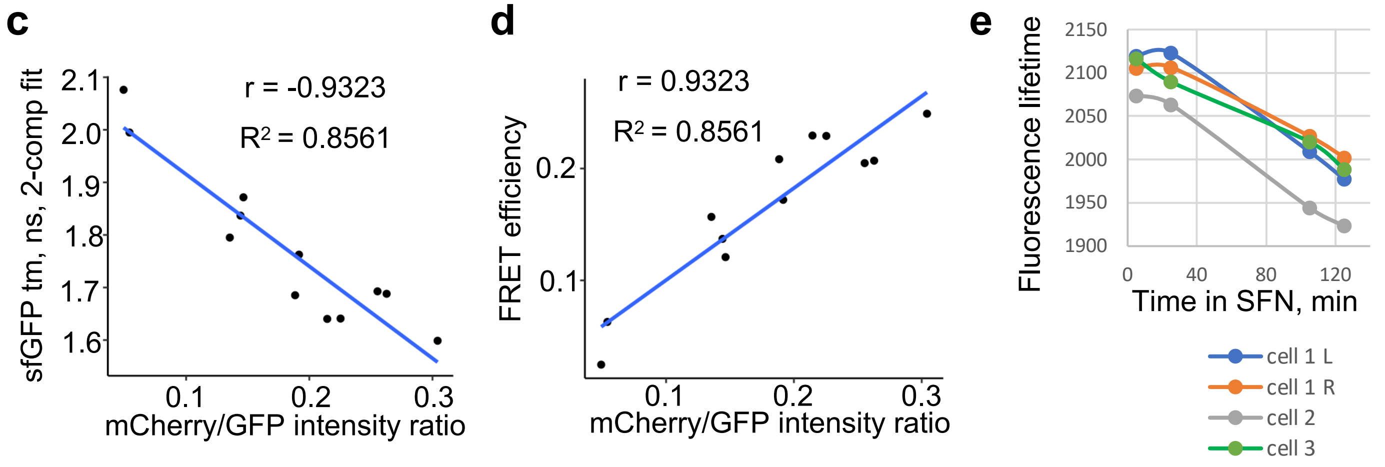

Supplementary Figure 2. Related to Figure 1 and 2. **Fluorescence lifetime analysis of FT-Nrf2.** **(a)** Analysis of the pooled photon trace data (black) from a representative measurement in **Figure 1f**, performed by fitting either 1-component exponential decay model ($F(t) = ae^{-t/\tau}$ [1], red) or 2-component exponential decay model ($F(t) = a(a1e^{-\frac{t}{\tau 1}} + a2e^{-\frac{t}{\tau 2}})$ [2] with $a1 + a2 = 1$ and fixed $\tau 2$ value, blue). The plot shows cumulative number of photons excited by repeated ultra-short pulses of laser illumination plotted against photon arrival time relative to the start of the corresponding pulse. The residuals for 1-component (red) or 2-component (blue) exponential fitting are shown on the bottom. The first 1.9 nsec of trace that include signal generated by the equipment (so-called Instrument Response Function) was removed from the analysis. The calculated goodness of fit (R^2) for each model is shown. Table shows fitted parameters and goodness of fit for each model as well as statistical significance (Pr(>F) value) of the difference between the two models calculated by ANOVA. **(b)** Quantification of datasets represented in Figure 1e. Fluorescence lifetime (tm) was quantified using 1-component analysis of the entire outlined cytoplasm (c) or nucleus (n). Statistical significance determined by t.test is indicated by * ($0.01 < p < 0.05$), or ns (for non-significant). (**c-d**) Strong (anti-)correlation with high goodness of fit between fluorescence lifetime determined by a 2-component exponential decay analysis (**c**) or FRET efficiency (**d**) and the mCherry/GFP intensity ratios in HeLa cells transfected with sfGFP-mCherry-Nrf2 shown in **Figure 1f**. Larger component in 2-complonent decay model was fixed at 2.1287, the value of FRET donor measured in cells transfected with sfGFP-Nrf2. **(e)** Quantification of sfGFP fluorescence lifetime in entire cellular areas of HeLa cells co-expressing FT-Nrf2 and Keap1 over the125 min course of treatment with 5 μM SFN shown in **Figure 2d**.

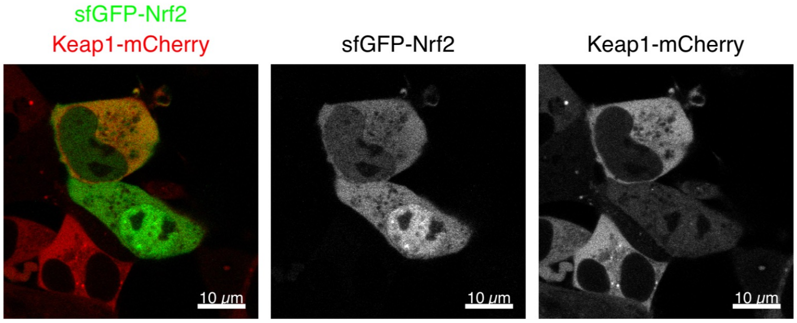

Supplementary Figure 3. Variability in Keap1 and Nrf2 expression in transient co-transfections. Related to **Figure 3.** Variability among HEK293 cells co-transfected with sfGFP-Nrf2 (middle grey panel and green on overlay) and Keap1-mCherry (right grey panel and red on overlay) for 1 day, imaged using confocal microscopy

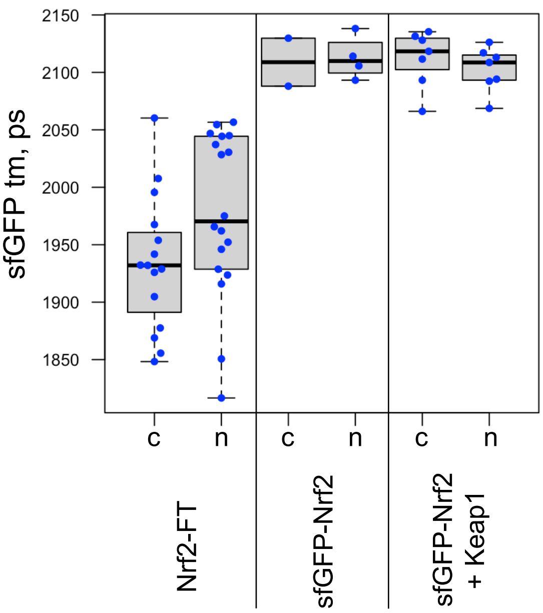

Supplementary Figure 4. Related to Fig 4. Quantification of datasets represented in Figure 4b. Fluorescence lifetime (tm) was quantified using 1-component analysis of the entire outlined cytoplasms (c) or nuclei (n). Nrf2-FT dataset is the same as ”WT” in Fig 4d, and sfGFP-Nrf2 dataset is the same as sfGFP-Nrf2 in Suppl. Fig. 2b.

Full-size images of the blots shown in Figure 2c and Figure 3b

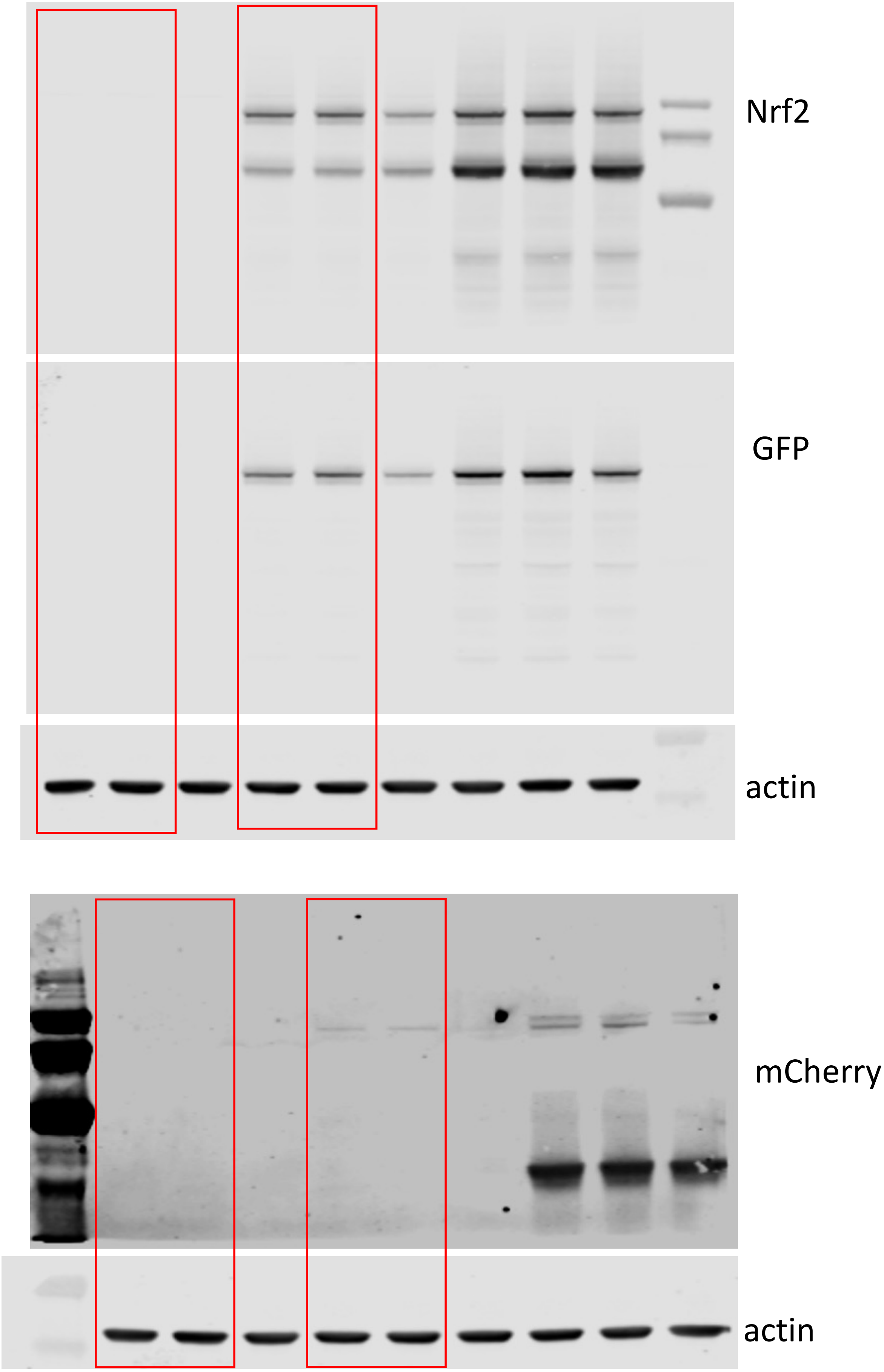

Full-size images of the blots shown in Figure 2c

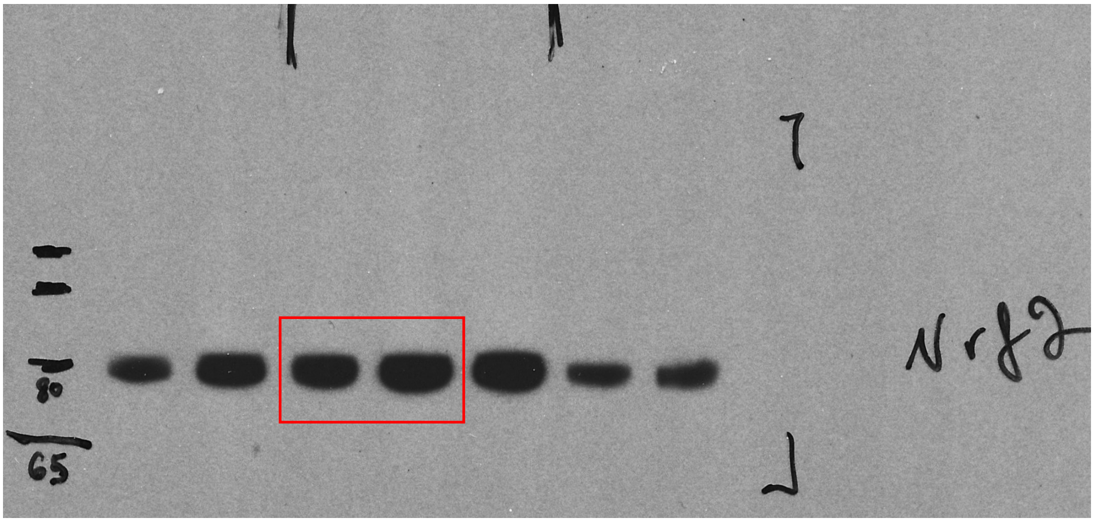

Nrf2
endogenous

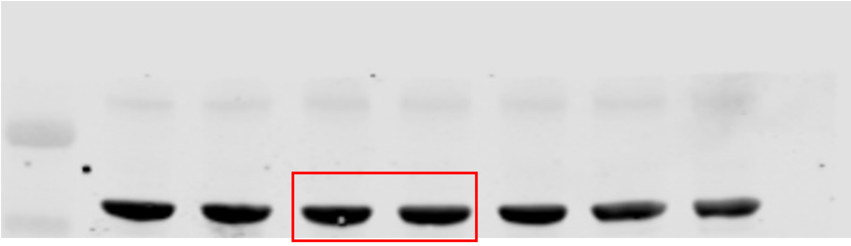

actin

Full-size images of the blots shown in Figure 2c, cont.

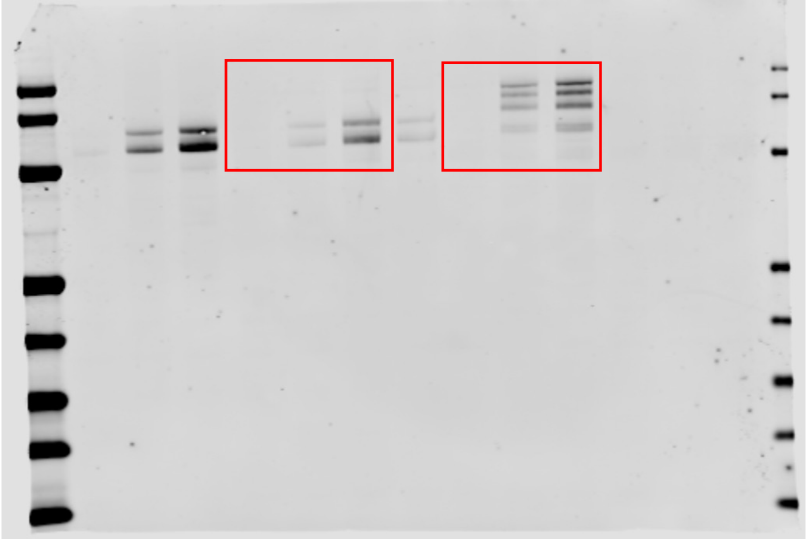

Nrf2

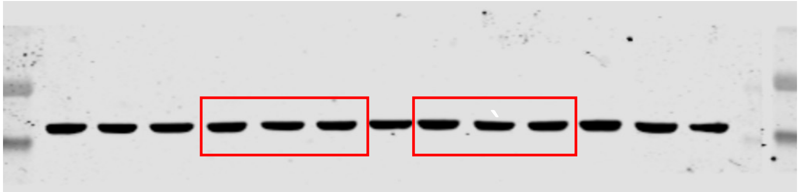

actin

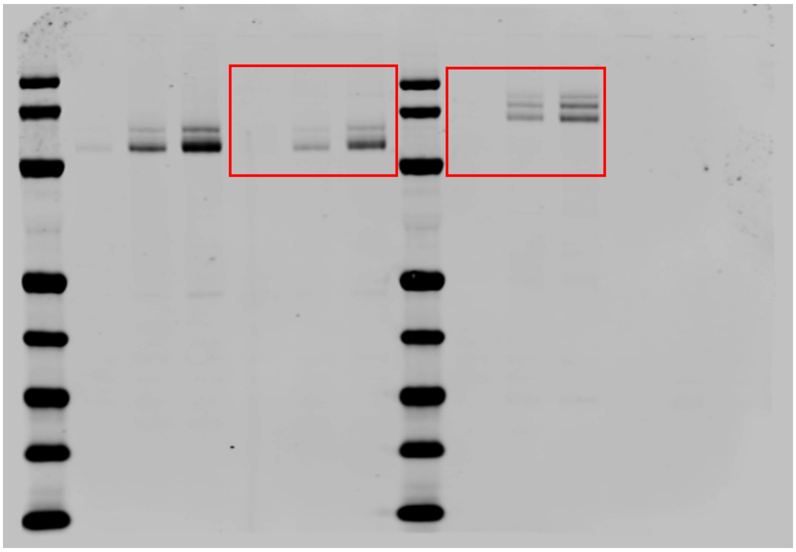

GFP

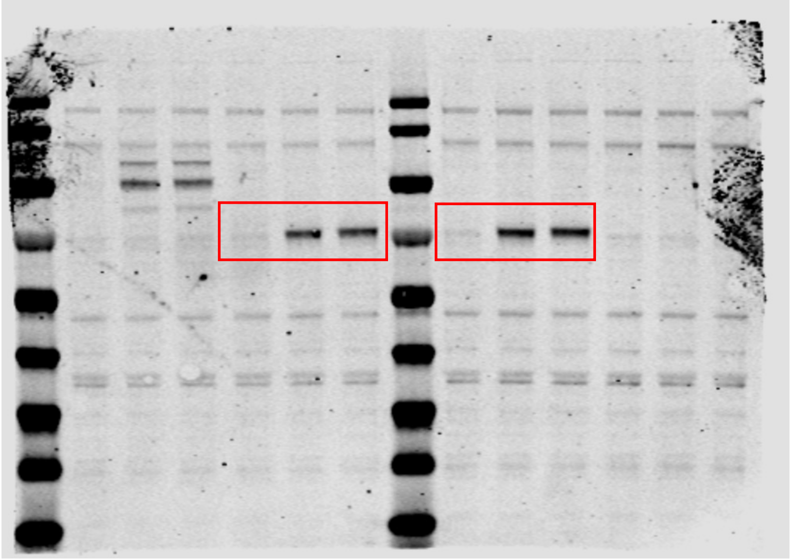

Keap1

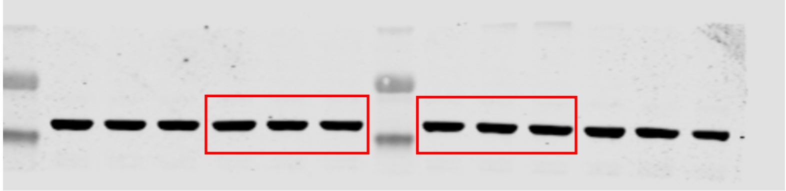

actin

Full-size images of the blots shown in Figure 3b
